# Case Report: Coexistence of bullous pemphigoid and psoriasis: Therapeutic challenge and IL17A-targeted parallel treatment strategy

**DOI:** 10.3389/fmed.2023.1148660

**Published:** 2023-04-03

**Authors:** Kossara Drenovska, Elia Valeva, Martin Shahid, Snejina Vassileva

**Affiliations:** Department of Dermatology and Venereology, Medical University - Sofia, Sofia, Bulgaria

**Keywords:** bullous pemphigoid, psoriasis, IL17A, secukinumab, biologics, treatment strategies

## Abstract

Autoimmune blistering diseases of the skin have all been reported in patients with psoriasis, bullous pemphigoid (BP) being the most frequently observed. The pathophysiologic triggers for BP in psoriatic patients are unclear. Recent observational studies have suggested that chronic psoriatic inflammation may cause pathological changes to the basement membrane zone, thus inducing autoimmunity against BP antigens through cross reactivity and “epitope spreading.” The coexistence of BP and psoriasis poses challenging therapeutic dilemmas related to the incompatibility of their standard treatments. Considering the probable common immunologic mechanisms in the pathogenesis of these inflammatory skin disorders, a suitable treatment regimen should be applied for their parallel control. We report three patients, who developed BP in the course of preceding long-lasting psoriasis. Secukinumab was administered as first-line treatment with promising therapeutic effect for both skin disorders and long-term disease control in two of the cases. In the third case, parallel disease control was initially achieved with methotrexate. A few years later, secukinumab was used for the treatment of a relapse of both dermatoses but worsening of BP was observed and methotrexate was reintroduced. Our experience on the therapeutic potential of secukinumab in BP is supported by the data in the literature. Recently, it was demonstrated that the proinflammatory cytokine IL17A has a functional role in the process of skin inflammation in BP, similarly to psoriasis. IL17A inhibition has emerged as a promising therapeutic strategy in patients with extensive or refractory BP but paradoxical development of BP after secukinumab treatment for psoriasis has also been described. This controversy emphasizes the need for further investigation into the development of optimal treatment strategies and recommendations.

## Introduction

Psoriasis is a chronic immune-mediated inflammatory skin disorder affecting 1–3% of the general population worldwide (1). Its pathogenesis is multifactorial, including environmental, genetic, and immune-related factors, triggering abnormal immune-mediated response involving the tumor necrosis factor α (TNF-α)/interleukin (IL)-23/IL-17 pathway (2–4). Psoriasis may be associated with cardiovascular or metabolic syndromes, chronic kidney disease, psoriatic arthritis (5), but also with some autoimmune blistering diseases (AIBDs), namely those of the pemphigoid group (6, 7). Bullous pemphigoid (BP) is an AIBD primarily affecting elderly patients and characterized by the presence of autoantibodies against two hemidesmosomal proteins, the transmembrane BP antigen 180 (BP180, collagen XVII) and the intracellular BP antigen 230 (BP230) (8). Likewise psoriasis, BP can be triggered by various physical (thermal burn, radiotherapy, ultraviolet light) and chemical factors, or multiple drugs, including topical and systemic antipsoriatic agents, or phototherapy (9–11). A recent large-scale population-based study has confirmed a bidirectional association between psoriasis and BP (12). Although the exact pathomechanism of this comorbidity is not fully elucidated, similarly to psoriasis, several *in vitro* and *in vivo* data have suggested the substantial role of IL-17 in the pathophysiology of BP (13–19). In this context, since 2017 we treated three patients with long-lasting psoriasis and recent-onset BP with the monoclonal anti-IL-17A antibody secukinumab (Cosentyx^®^, Novartis, Basel, Switzerland) and report our therapeutic experience.

## Case 1

**FIGURE 1:**
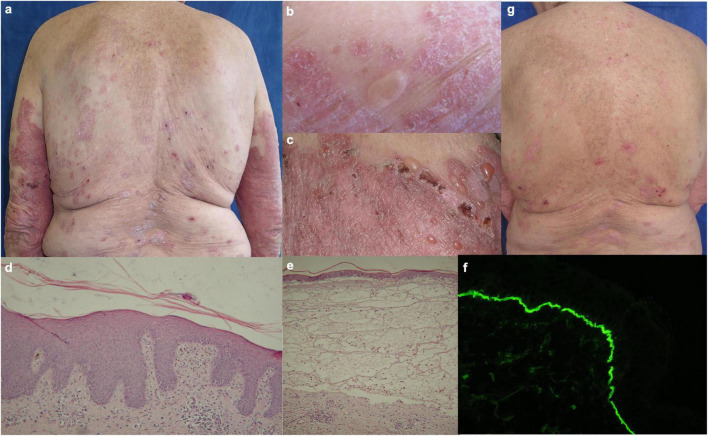
Clinical, histological and immunofluorescent findings in patient 1: **(a–c)** Figurate and confluent psoriatic plaques with multiple tense blisters and crusted erosions on healthy and psoriatic skin; **(d)** histology from psoriatic plaque with abundant eosinophil infiltrate; **(e)** subepidermal clefting with abundant mixed infiltrate in papillary dermis and eosinophil predominance interstitially; **(f)** direct immunofluorescence with linear deposits of IgG along the BMZ in perilesional skin; **(g)** reduction of erythema, desquamation and blistering during secukinumab treatment.

A 70-year-old Caucasian woman with a 25-year history of chronic plaque-type psoriasis was admitted to our dermatology department because of the acute onset of a pruritic blistering eruption a month before. Her medical history was relevant for arterial hypertension controlled with lisinopril, moxonidine, and ivabradine. Throughout the years, her psoriasis was maintained with topical agents, phototherapy, and multiple methotrexate courses up to a total cumulative dose of 2.8 g. Upon admission, a flare of psoriasis was revealed with figurate and confluent psoriatic plaques over the trunk and extremities. Alongside, multiple tense blisters and crusted erosions were observed over normal appearing and psoriatic skin (Figures 1a–c).

The routine laboratory was unremarkable except for an elevated erythrocyte sedimentation rate (ESR), normochromic anaemia, and peripheral eosinophilia. Histopathology examination of biopsy specimens from bullous and psoriatic lesion was compatible with subepidermal bullous dermatosis and psoriasis, respectively (Figures 1d, e). Direct immunofluorescence (DIF) on peribullous skin demonstrated linear IgG and C3 complement deposits along the basement membrane zone (BMZ) (Figure 1f). Indirect immunofluorescence (IIF) on monkey esophagus detected serum IgG anti-BMZ antibodies with a titer of 1:640, reactive with the roof of monkey salt split skin (SSS) substrate. Both anti-BP180-NC16A and anti-BP230 ELISA (Euroimmun, Lübeck, Germany) were strongly positive, over 200 RU/ml and 96 RU/ml, respectively (cut-off value 20 RU/ml). Based on these data the diagnosis of BP was confirmed.

Treatment with secukinumab was introduced with a loading dose of 300 mg s.c. at weeks 0, 1, 2, 3, and 4, followed by 300 mg every 4 weeks. Patient’s Psoriasis Area and Severity Index (PASI), Bullous Pemphigoid Disease Area Index (BPDAI), and Dermatology Life Quality Index (DLQI) scores before and during secukinumab treatment are summarized in Table 1. Even at week 3, a reduction of pruritus, blistering, erythema, and desquamation was noted (Figure 1g) followed by a complete remission of more than 5 years duration for both psoriasis and BP. Recent ELISA follow-up demonstrated positive anti-BP180 and anti-BP230 results, 59 RU/ml and 163 RU/ml respectively, while the patient remained in continuous clinical remission under secukinumab therapy.

**TABLE 1:** Therapeutic effect of secukinumab on psoriasis and BP disease activity and quality of life in the three reported patients.

			Before secukinumab therapy	One month after secukinumab therapy
Case 1	BPDAI	Erosions/blisters	50	17
		Urticaria/erythema	23	13
		Mucosal involvement	0	0
		Total	73	30
	PASI		43.3	0.2
	DLQI		24	10
Case 2	BPDAI	Erosions/blisters	39	72
		Urticaria/erythema	21	50
		Mucosal involvement	0	0
		Total	60	122
	PASI		20.2	0.4
	DLQI		10	21
Case 3	BPDAI	Erosions/blisters	47	3
		Urticaria/erythema	30	6
		Mucosal involvement	0	0
		Total	77	9
	PASI		23.1	0.2
	DLQI		23	3

PASI, psoriasis area severity index; BPDAI, bullous pemphigoid disease area index; DLQI, dermatology life quality index.

## Case 2

**FIGURE 2:**
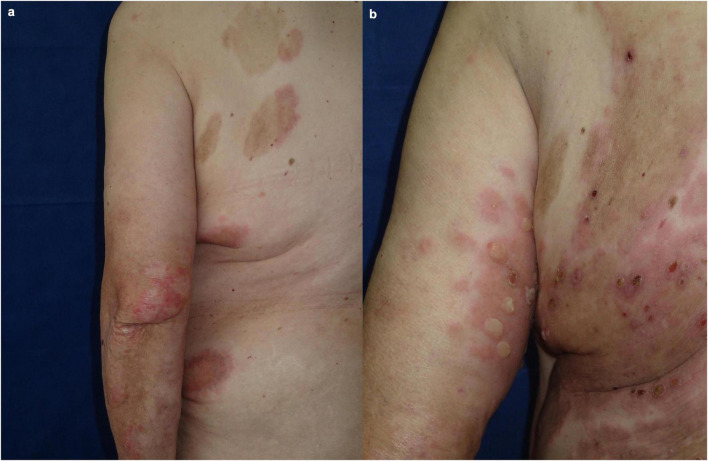
Clinical presentation of patient 2: **(a)** Flare of both psoriasis and BP while on reduced dose of methotrexate; **(b)** worsening of BP during secukinumab treatment.

A 71-year-old Caucasian woman with a 5-year history of psoriasis vulgaris was initially admitted to our department for a generalized pruritic blistering eruption following a course of spa- and quartz lamp procedures for a flare of her psoriasis. Additionally, the patient suffered from arterial hypertension controlled by amlodipine, valsartan, and clonidine hydrochloride. Laboratory tests revealed leukocytosis, elevated serum creatinine, and blood urea nitrogen. The histology of a bullous lesion was compatible with BP. DIF on peribullous skin revealed linear deposition of IgG and C3 along the BMZ. Epidermal pattern serum IgG BMZ antibodies at a titer of 1:320 were found by IIF on SSS substrate. The diagnosis of BP was further supported by positive ELISA BP180 exceeding 200 RU/ml whereas ELISA BP230 was negative. Treatment with systemic corticosteroids was avoided due to the concomitant psoriasis. Methotrexate 15 mg/weekly with subsequent tapering, along with topical corticosteroids and emollients led to good control of both skin disorders. Six years later, the patient returned with relapse of both psoriasis and BP (Figure 2a). Based on our previous experience, secukinumab was administered at a conventional regimen with a loading dose of 300 mg/weekly for 4 weeks. In the course of the first month of secukinumab application, worsening of BP was observed with aggravation of the subjective complaints and appearance of multiple new bullae on erythematous background, while psoriatic lesions healed almost completely (Figure 2b). Secukinumab was discontinued and methotrexate was reintroduced at a dose of 5 mg/weekly resulting in parallel control of both skin diseases (Table 1).

## Case 3

**FIGURE 3:**
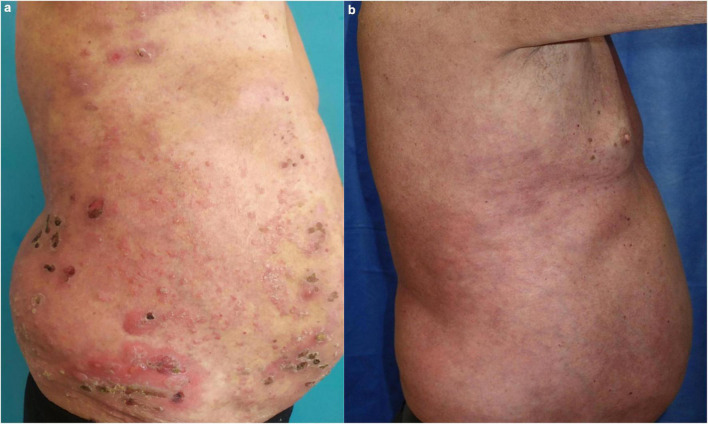
Clinical presentation of patient 3: **(a)** Diffuse scaly erythema on the trunk and extremities with superimposed multiple tense vesicles and bullae on both healthy and psoriatic skin; **(b)** complete control of both psoriasis and BP during secukinumab treatment.

A 65-year-old Caucasian man was hospitalized for a widespread, pruritic blistering eruption of three weeks duration. The patient had history of plaque-type psoriasis of 30-years duration, treated over the years with topical steroids, emollients, photo- and thalassotherapy, as well as a course of methotrexate. Due to a recent flare of his psoriasis the patient exposed himself extensively to the sun while gardening, which was followed by the appearance of disseminated blisters. Dermatological examination upon admission revealed diffuse scaly erythema on the trunk and extremities with superimposed multiple tense vesicles and bullae on both healthy and psoriatic skin (Figure 3a). Laboratory tests demonstrated increased ESR and C-reactive protein, peripheral eosinophilia, and elevated gamma-glutamyl transferase. Histopathology of biopsy specimens from a psoriatic plaque and the edge of a blister was compatible with psoriasis and BP, respectively. DIF on peribullous skin demonstrated linear IgG and C3 along the BMZ. Positive ELISA BP180 of 70 RU/ml and negative ELISA BP230 confirmed the diagnosis of BP. Systemic corticosteroids were avoided due to the underlying psoriasis. Topical emollients and potent corticosteroids led to slight improvement. Secukinumab at initial weekly administration of 300 mg during the first month and subsequent monthly application achieved complete and stable control of both skin disorders for the next 1.5 years (Figure 3b and Table 1). ELISA follow-up for anti-BP180 and anti-BP230 was negative, in the context of continuous 18-months clinical remission under secukinumab treatment.

## Discussion

The association between psoriasis and a bullous eruption has been first described almost a hundred years ago (20). Later, psoriasis has been reported to coexist with pemphigus (21), cicatricial pemphigoid (22), and epidermolysis bullosa acquisita (23), but BP remains the most commonly associated immunobullous disease (7, 24–26). In addition, one-third of the cases of anti-laminin gamma1 or p200-pemphigoid are associated with psoriasis (27, 28).

The pathomechanisms underlying this association are likely driven by autoimmune processes but their precise nature remains unknown (25). The implication of T-helper 17 (Th17) cells and the production of IL-17A/F cytokines is a plausible pathogenic link between both diseases that has led to a paradigm shift in their treatment.

Psoriasis immune response is mainly driven by IL-17 producing T-lymphocytes (T17) (29) of both CD4 + (Th17) and CD8 + T-cytotoxic 17 (Tc17) subsets (30–33). Dysregulation between T17 and T regulatory (Treg) cells in the skin promotes inflammatory responses that result in abnormal proliferation of keratinocytes and extensive infiltration of inflammatory cells. IL-17 is regarded as the direct regulator that stimulates keratinocyte proliferation and inhibits keratinocyte differentiation (34).

BP is primarily a Th1/Th2 cell-mediated disease with predominance of Th2 response and production of BP180/BP230 autoantibodies (16). Binding of autoantibodies to their target antigens results in complement activation, mast cell degranulation, polymorphonuclear infiltration, and release of enzymes such as proteases and elastases that induce dermal-epidermal separation (35). Recently, the participation of Th17 cells in the exacerbation of the BP inflammatory response was highlighted, which together with dysregulated regulatory T cells (Tregs) promote the activation of autoreactive T cells and autoantibody production. Both Th17 and Treg cells are elevated in BP affected skin as shown by immunohistochemical studies (14–18). Additionally, detection of increased IL-17 levels in BP blister fluid is suggestive for its key role in the eosinophilic infiltration and consequent BMZ damage (13, 14). Inhibition of IL-17A has been shown to prevent dermal–epidermal separation in cryosections of normal human skin incubated with anti-BP180 IgG and leukocytes pre-treated with anti-IL-17A IgG (18). In the passive antibody transfer mouse model, a correlation was found between the serum levels of IL-17A and the severity of lesion formation in mice. In addition, the same study has shown that IL-17A-deficient mice are protected against induction of experimental murine BP, and pharmacological inhibition of lL-17A significantly reduces the extent of skin lesions (18). Finally, a long-term remission of severe BP along with clearance of the circulating IL-17A-positive CD4 + cells and anti-BP180 antibodies has been achieved after secukinumab treatment (36). Surprisingly, a recent clinical trial of ixecizumab, another anti-IL-17A biologic agent approved for the treatment of psoriasis, failed to achieve the primary and secondary endpoints in the treatment of BP (NCT03099538) (37).

Taken together, all these data point out to IL-17 as a candidate target molecule in the treatment strategy of coexistent psoriasis and BP, which was quite challenging in the past. It is well known that systemic corticosteroids indicated for BP (38), together with tertracyclines may aggravate concomitant psoriasis (39–42). Cytotoxic agents such as methotrexate, azathyoprine, mycophenolate mofetil, and cyclosporin are reasonable alternatives but the latter two might be inaccessible because of their high price (43, 44). Dapsone (45) has been occasionally reported to successfully treat the blistering eruption but with regard to psoriatic lesions it is considered as appropriate in pustular psoriasis mainly (46). Finally, most physical modalities (UVB, psoralen UVA) or topical antipsoriatic agents may induce bullous lesions (22).

In recent years, biological agents including TNF-α, IL-17, IL-12/23, and IL-23 inhibitors, have revolutionized psoriasis treatment due to a long-term efficacy and highly favorable safety profile (47). Notably, the effectiveness and early onset of skin clearance of IL-17A inhibitors was confirmed in both randomized clinical trials and in the real-world setting (48).

Published data on successful biologic therapy of concomitant BP and psoriasis include one case treated with etanercept (49), one with ustekinumab (50), two cases with ixekizumab (51, 52), and two cases with secukinumab in combination with prednisolone (53, 54).

In our three patients secukinumab was administered as monotherapy and was the treatment of choice as no other IL-17A inhibitor was available in Bulgaria at the time of our first clinical observation, the latter being initially presented at a national conference (55). Secukinumab alone, combined only with topical emollients and keratolytics achieved complete BP remission, as well as progressive clearance of the psoriatic lesions in two of the three patients, cases 1 and 3. Contrary to this good experience, our second patient had no effect and even worsening of BP was observed, so methotrexate was readministered. Similar scenario is not an exception as paradoxical BP onset following secukinumab treatment for psoriasis has previously been described (56).

All three reported patients demonstrated high titers of anti-BP180 at the time of active BP, the first one being also positive for anti-BP-230. Serological follow up of both patients successfully treated with secukinumab revealed controversial results. Patient 1 preserved positivity for both autoantibodies although a pronounced decrease of anti-BP180 was observed, while anti-BP230 has been found slightly elevated. On the opposite, a complete lack of antibodies against BP180 and BP230 was found in case 3. This dynamics in the immune serologic profile coincided with a continuous clinical remission in both patients who remained without known relapses under secukinumab therapy. Despite this controversy, our data correspond to previous reports where serum levels of anti-BP180 antibodies have been found to decrease after treatment with secukinumab although in combination with systemic steroids (53). The exact mechanism of this decrease remains unclear but previous serological studies on IL-17 and IL-23 levels followed by experimental mouse models of BP have demonstrated increased IL-17 expression in lesional skin, serum, and blister fluid, which contributes to blister formation through activation of neutrophils (17, 18).

The reported studies have drawn attention to the possible targeting of IL-17 in BP and have revealed the therapeutic potential of anti-IL-17 therapy as a promising therapeutic approach. In this regard, there might be a rationale for performing screening tests for the activation of the IL17/IL23 axis before the administration of secukinumab. To our knowledge, our three reported patients are the only ones treated with secukinumab alone.

## Conclusion

Chronic plaque-type psoriasis is dominated by the Th-17/IL-23 axis but there is also a vast amount of data supporting the role of IL-17 in the pathogenesis of BP. This reveals potential for novel therapeutic approaches, namely the IL-17A inhibition that presents promising therapeutic strategy in patients with coexistent psoriasis and BP. Despite the presence of many supportive data, high-quality studies are lacking and are needed to better clarify the optimal treatment modalities or to explain the eventual therapeutic pitfalls in such associations.

## Data availability statement

The original contributions presented in this study are included in the article/supplementary material, further inquiries can be directed to the corresponding author.

## Ethics statement

Ethical review and approval was not required for the study on human participants in accordance with the local legislation and institutional requirements. The patients/participants provided their written informed consent to participate in this study. Written informed consent was obtained from the participant/patient(s) for the publication of this case report.

## Author contributions

All authors listed have made a substantial, direct, and intellectual contribution to the work, and approved it for publication.
